# Management of Intracanal Separated File Fragment in a Four-Rooted Mandibular Third Molar

**DOI:** 10.1155/2021/5547062

**Published:** 2021-06-30

**Authors:** Benoy Jacob, Anjaneyulu K., Aishwarya Ranganath, Riluwan Siddique

**Affiliations:** Department of Conservative Dentistry and Endodontics, Saveetha Institute of Medical and Technical Sciences, Chennai, Tamil Nadu, India

## Abstract

The success of endodontic therapy is attributed to complete arbitration of the bound entities concealed within the complexity and absolute disinfection of the root canal system, thus, deeming it mandatory to effectively negotiate and overcome the challenges posed by obstruction, either iatrogenic or anatomic. To achieve this, considerable depth of knowledge and expertise with reference to variations in root canal morphology and clinical mishap management is substantially as important as developing fine observation skills in conjunction with an appropriate armamentarium and a keen sense of determination, thereby enhancing one's clinical acumen by several folds. In the present case, following rubber dam isolation, the temporary restoration was removed, and the remaining carious dentin was excavated. Endodontic access cavity was refined and explored with a DG-16 probe, following which three separate canal orifices were identified in the pulp chamber floor (mesiobuccal, mesiolingual, and distal). On further observation under a surgical operating microscope and continuous exploration with the DG-16 probe, a fourth canal was found in the mesial aspect of the tooth (middle mesial). With instrumentation, it was confirmed that a fractured object was indeed present at the apical third of the mesiolingual root of tooth 38. Bypassing of the fractured fragment was initiated with a size 10 SS K-file coupled with copious irrigation with 3% sodium hypochlorite. In the present case report, four distinct canals comprising 3 mesial and 1 distal canal were recognized, and the fractured instrument in one of the canals was bypassed successfully.

## 1. Background

The root canal in all its glory is a relentless enigma unfolding itself during the endodontic treatment procedure. It is therefore pertinent to remember that a complete understanding of the bound entity is indeed an essentiality in order to achieve the most prolific of all outcomes [1]. A successful endodontic treatment is immeasurably attained through persistent chemo-mechanical cleansing of the root canal system [2]. The above statement is adjudged for the very fact that the interplay between an in-depth knowledge of tooth anatomy and root canal morphology conjoined with meticulous planning for the proposed treatment is often necessary to rid the root canal complexities of microorganisms and pulp tissue remnants [1]. Aberrations in anatomy are sizeably as important as the normal anatomy of the root canal system, thereby strengthening the need to acquire considerable depth of knowledge and expertise in this regard [1, 3].

The general prerequisites to be considered prior to the management of a complex anomaly often involve earlier discussed criteria as well as a definite focus on the pulp chamber floor, proper judgement, and use of sufficient armamentarium keeping in mind the biological anthropology of a tooth and its varied anatomy [4]. Apart from the usual and aberrant configurations concerning permanent first and second molar teeth, wide variations in the root canal morphology with respect to permanent third molars have also been studied and investigated upon [5]. Although in vitro studies, by clearing methods, have established the incidence of variations in the number of canals in mandibular third molars [6–9], however, there exists scarcity in clinical cases with advanced imaging modalities. This paper brings to the fore the case of a successful nonsurgical clinical aberrative and mishap management of a previously initiated four-rooted left mandibular third molar harbouring four root canals whose configuration of 3 mesial and 1 distal root was confirmed following CBCT evaluation, with a 1-year follow-up for the same.

## 2. Case Description

**Figure 1:**
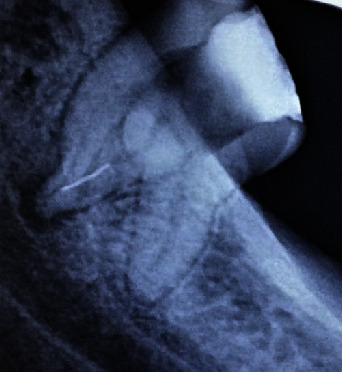
Preoperative radiograph.

A 31-year-old Indian male patient reported to the Department of Conservative Dentistry and Endodontics with a chief complaint of spontaneous pain in the lower left back tooth region for the past 4 days. Medical history was noncontributory. The patient revealed a history of intermittent pain for 3 weeks which continued to increase in severity, aggravated while chewing food at the affected site, and relieved with medication. The patient gave a detailed history on the subjective symptoms and disclosed that the tooth had been treated 8 months back. On clinical examination, it was noticed that the patient had undergone extraction of teeth 36 and 37, and restoration was present in 38. Soft tissue examination revealed neither oedema nor tenderness to palpation. The tooth elicited moderate to severe tenderness on the application of finger pressure. Multiple parallax radiographs were taken to assess the condition of the tooth and its surrounding structures. The preoperative radiograph revealed radiopacity in the pulpal chamber and at the apical third of one of the roots (Figure 1). While the former was indicative of a restorative material, the latter on the other hand disclosed a fractured/separated instrument from the previously initiated therapy (Figure 1). Furthermore, the periapical region of the said tooth revealed a radiolucent area. Based on both clinical and radiographic findings, a diagnosis of previously initiated, symptomatic apical periodontitis, was confirmed. Nonsurgical endodontic therapy was proposed as the treatment of choice.

**Figure 2:**
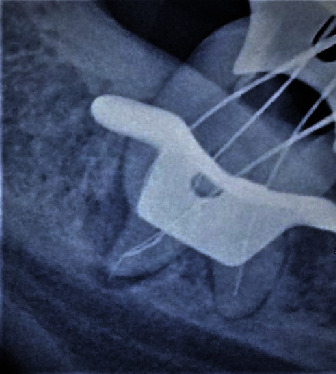
File bypass/working length.

The tooth was anaesthetised with 1.8 ml 2% lignocaine containing 1 : 200,000 epinephrine (Xylocaine; AstraZeneca Pharma India Ltd., Bangalore, India). Following rubber dam isolation, the temporary restoration was removed, and the remaining carious dentin was excavated. The endodontic access cavity was refined and explored with a DG-16 probe (Hu-Friedy, Chicago, IL, USA), following which three separate canal orifices were identified in the pulp chamber floor (mesiobuccal, mesiolingual, and distal). On further observation under a surgical operating microscope (Carl Zeiss OPMI Pico, Jena, Germany) and continuous exploration with the DG-16 probe, a fourth canal was found in the mesial aspect of the tooth (middle mesial). Once all four canals were scouted, the access was modified into a trapezoidal cavity using an Endo Z bur (Dentsply Maillefer, Baillagues, Switzerland) to obtain straight-line access of all four canals. Nickel-titanium (NiTi) ProTaper Gold SX orifice shapers (Dentsply Maillefer) were used for coronal enlargement followed by patency establishment using a size 10 stainless steel (SS) K-file (Dentsply Maillefer). With instrumentation, it was confirmed that a fractured object was indeed present at the apical third of the mesiolingual root of tooth 38. Bypassing of the fractured fragment was initiated with a size 10 SS K-file coupled with copious irrigation with 3% sodium hypochlorite. The size 10 K-file was implemented mesially along the inner curvature of the target site to bypass the fragment lodged within the curvature. Following each attempt, the file was removed, inspected, cleaned, and engaged again with a gentle push-pull and/or watch winding motion. On inspection, if a file was distorted, it was immediately replaced with a new size 10 K-file and instrumented repeatedly as described above till the resistance was bypassed, after which a size 15 SS K-file was introduced and worked in a similar fashion. Subsequent radiographs were taken to understand the extent of bypassing of the fractured fragment achieved with the size 15 K-file. Once the fractured file was bypassed completely (Figure 2), sequentially larger files (size 20 K-files) were introduced seamlessly till the size 25 SS K-files working their way to the apex, as described earlier. It is to be noted that the entire bypassing procedure was facilitated with a 3% sodium hypochlorite solution as a lubricant. Next, working length determination was performed using an electronic apex locator (Root ZX; J Morita, Tokyo, Japan) and was established at 18 mm for the distal canal, 17 mm for the mesiolingual and mesiobuccal canals, and 16 mm for the middle mesial >canal. Then, the remainder of the canals was apically prepared up to size 25 SS K-file, further shaping and finishing was achieved with ProTaper Gold (Dentsply Maillefer) NiTi rotary instrumentation (sequentially) using an electric motor (X-Smart, Dentsply Maillefer) along with subsequent recapitulation per sequence. Final cleansing of the canals was achieved with 17% EDTA solution (META BIOMED CO.LTD), intermediate flushing with normal saline followed by 3% sodium hypochlorite as the endmost irrigant. Canals were dried with absorbent paper points (Dentsply Sirona) after which a thick creamy mix of calcium hydroxide powder mixed with 3% sodium hypochlorite [10, 11] was incorporated into the canals with lentulospirals (Dentsply Maillefer). Cotton pellet was placed, and the access cavity was temporarily restored with Cavit. The patient was prescribed dolonex 20 mg tab DT twice daily for two days for relief of painful symptoms, and the next appointment was scheduled after two weeks.

**Figure 3:**
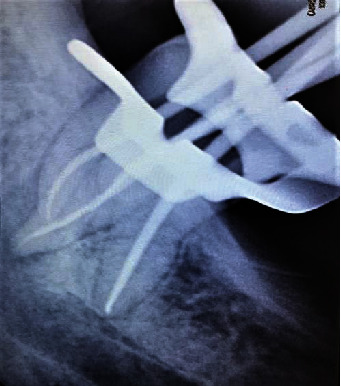
Master cone.

**Figure 4:**
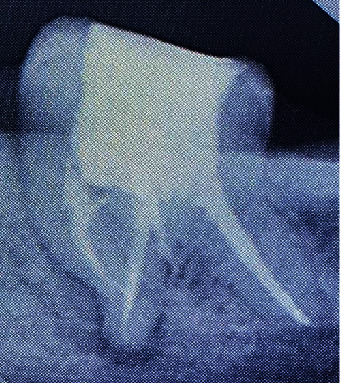
Postoperative radiograph.

**Figure 5:**
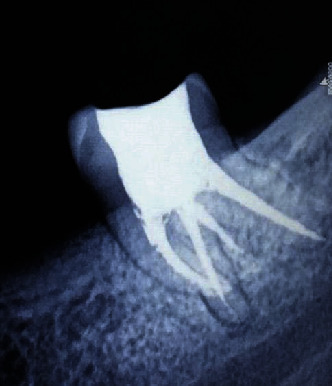
1-year follow-up.

At the second appointment, the patient was asymptomatic with resolution of signs and symptoms. After removal of the temporary restoration along with intracanal dressing, care was taken to flush out the remaining medicament from the canal system by way of copious saline irrigation and 3% sodium hypochlorite. Sonic agitation was executed using the EndoActivator® with a 25/04 noncutting polymer tip (Dentsply Specialities, Tulsa, OK, USA). Following irrigant delivery, the tip was placed 1 mm short of the working length for each canal, activated for 30 seconds, and then removed. Drying of the canals was made certain with absorbent paper points (Dentsply Sirona), following which the master cone fit was checked and confirmed on a radiograph (Figure 3). Obturation of the prepared canals was attained by the single cone gutta-percha (Dentsply Maillefer) technique with AH Plus resin-based sealer (Dentsply DeTrey, Konstanz, Germany) as the sealant (Figure 4). The tooth was then restored with a Filtek Z350XT (3MESPE) posterior composite resin material. The patient was followed up at regular intervals. At 1-year follow-up, a radiograph was taken (Figure 5) and a CBCT scan was performed. CBCT (Dentsply Sirona, Orthophos XG 3D) was taken at standardized settings (90 kV, 6 mA, 5∗5.5 cm, 160 *μ*m,14 s). Reduction in size of the lesion was markedly visible at the 1-year follow-up period (Figure 5).

## 3. Discussion

The mandibular third molar is the last tooth to erupt into the oral cavity, usually between 17 and 21 years of age or at a later stage in life, partially erupt or perhaps not erupt at all [12]. Owing to its nonfunctional position, much credence has been given to early uprooting of the third molar tooth to rid the patient and operator of the diverse problems in dealing with it [13, 14]. However, current practice asserts “to serve, save and protect” the functional tooth at any cost, and the best way to save it is “to not lose it at all” in the first place. The retention of the third molar in clinical situations wherein the opposing tooth is either present or missing does not hold significance in this case as the abutting second molar was absent. Therefore, in the present case, root canal therapy was perpetrated to conserve the tooth in question, consequently serving as a prudent abutment for a possible prosthesis [13]. Most importantly, despite being treated upon earlier by another practitioner, the patient reported continuous nagging pain partly because of it and partly due to the patient's inability to return to the previous practitioner for the remainder of the treatment. The patient's desire to retain the said tooth was prioritized and primarily taken into consideration.

The mandibular third molar is indeed a cut above the rest with reference to anatomical variation [15]. Barring a few infrequently reported anatomical occurrences, the solitary or binary variants entombing one to four canals are observed to be the most recurrent [6, 7, 16]. Silberman et al., 2009, reported a peculiar case of a three-rooted mandibular right third molar tooth encasing 5 canals (4 mesial, 1 distal), for which endodontic therapy was successfully accomplished [17]. Apart from the above published clinical case, two studies incorporating in vitro clearing methods confirmed the presence of 5 canals in similar teeth [6, 7]. Plotino, in 2008, was the first to report a rare clinical case wherein three mesial canals including a distal canal were identified in a four-rooted mandibular third molar tooth [18]. Most conspicuously, Sinha and Sinha, 2014, in their case report, described the management of a mandibular third molar housing 5 canals, thus, contributing to the literature significantly [19]. Periapical radiograph was the imaging modality of choice in both the above-described case reports. In the present case report, CBCT was not opted for at first as the canal orifices could be detected with the aid of a dental operating microscope (Carl Zeiss OPMI Pico, Jena, Germany) and a champagne bubble test. Moreover, the orifices first identified/located were almost equidistant from one another, in accordance with the laws of symmetry as proposed by Krasner and Rankow, 2004 [4].

At 1 year follow-up, it was decided that a CBCT scan should be done to highlight the unique four-rooted variation of the mandibular third molar, encasing four distinct canals [20, 21]. Additionally, an advanced radiographic modality regarding such an anomaly has not been employed in previously published case reports. Moreover, the extent of healing of the lesion involving the teeth could also be assessed with CBCT in addition to a conventional radiograph. According to the guidelines put forward by the American Association of Endodontics (AAE), CBCT scan with a small field of view is recommended in clinical research trials for diagnostic accuracy [22]. Furthermore, in the present case, much credence had been given to achieving a sterile environment, enforcing a stringent disinfection protocol pertaining to irrigant delivery, its activation, and placement of a suitable intracanal medicament. Time and again, the judicious application of calcium hydroxide as an interappointment medication for effectively controlling microbial activity in the root canal system has been authenticated. Interestingly, Hamed et al., 2014, concluded in their research that the combined antibacterial effect of the mixture of calcium hydroxide and sodium hypochlorite as intracanal medicament was superlative against both gram-positive and gram-negative bacteria in comparison to calcium hydroxide when used alone. The notable findings from the above-stated were in conformity with those produced in a study effectuated by Farhad et al., 2012 [23].

The most common site of occurrence of separated instruments has often been reported in mandibular molars. The aforesaid statement is reckoned from the fact that compelling evidence has been accorded for the same, as disclosed by Shen et al., 2004, Cujé et al., 2010, and Nevares et al., 2012 [24–26]. Interestingly, fractured instruments are claimed to not be the direct cause of recontamination emanating from the root canal complexity [27]. Furthermore, on a riveting note, it was affirmed in a systematic review by Panitvisai et al., 2010, that there was no statistically significant difference in healing rates between teeth with and without retained instrument fragment [28]. However, when a periradicular lesion is present, it becomes even more obligatory that the fragment is either removed or bypassed, thus, envisaging acceptable/satisfactory/complete healing of the infected tooth [26]. The success of endodontic therapy depends on complete disinfection of the root canal system, and its outcome solely depends upon the depth at which an irrigant effectively penetrates [29], thus, deeming it mandatory to effectively negotiate and overcome the challenges posed by obstructions, either iatrogenic or anatomic, within the root canal pathways. Various factors influence the outcome of the treatment in case of fractured fragments, namely, the root canal anatomy, presence of curvature, the position of the fractured instrument, and the ability of the operator to negotiate or retrieve the foreign object [30].

In routine dental practice, by and large, it is uncommon to frequently come across retained instrument fragments in the root canals of endodontically initiated and/or treated teeth with a low rate of incidence being reported for the same. However, during a serendipitous encounter, adept knowledge, and dexterous proficiency in this regard succour to assess the limitations in hand and opt for the best suitable technique coupled with existing and/or emerging technologies available, capable of ensuring a better prognosis for the tooth in question, as evinced in the present case report. With reference to root canal aberrancies, “there's more to it than meets the eye,” as the root canal is an incessant conundrum unto itself. Having said that, the acronym “KODAC”-sound knowledge (K) of aberrant anatomy, keen observation (O) skills for locating shrouded canals, ever-zealous sense of determination (D), indispensable armamentarium (A) for scouting, enduring commitment (C) to follow-up such cases, holds significant credibility in the long-term successful management of complex root canal anatomies and mishaps of iatrogenic/anatomical origin.

## 4. Conclusion

Existing literature reveals that the greatest percentage of instrument fracture is witnessed in the apical third of the root canal, probably due to inadequate patency and canal width [31–33]. At this juncture, negotiating the file can be considered initially as attempts to retrieve the fractured instrument may further result in the reduction of root dentin at the apical third, transportation, ledge formation, or perhaps even perforation [34, 35]. In the present case report, the fractured instrument was bypassed successfully.

**Figure 6:**
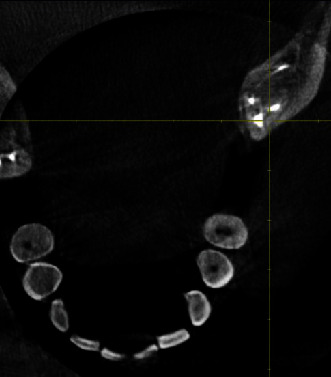
CBCT axial view (postoperative).

In the present case report, from a clinical and radiographic point of view, four distinct canals were recognized. Following a CBCT examination, 3 mesial and 1 distal canal (3 M and 1D) were clearly discernible (Figure 6). General inevitable discrepancies must be expected owing to certain anatomical anomalies such as those involving a tooth which has become rotated, subsequently contributing to confounding yet perceivable anatomical complexities by employing 3-dimensional diagnostic imaging modalities for diagnostic precision. Adding on to the existing literature, the present case report deals with a patient of Indian origin, thus, emphasizing the additional importance of attaining familiarity with variations in root canal anatomy and complexities of diverse ethnicities.

## Data Availability

Data are available on request.
